# Monitoring the implementation of the WHO Global Code of Practice on the international recruitment of health personnel: the case of Indonesia

**DOI:** 10.1186/1472-6963-14-S2-P35

**Published:** 2014-07-07

**Authors:** Ferry Efendi, Ching-Min Chen

**Affiliations:** 1 Institute of Allied Health Sciences, College of Medicine, National Cheng Kung University, Tainan, Taiwan; 2 Department of Nursing, National Cheng Kung University, Tainan, Taiwan

## Background

Indonesia has become one of the international nurse migration players that has supported the Code that was endorsed by the World Health Assembly, year 2010. In reference to the Code, the Minister of Health (MoH) as designated by the national authority, issued the regulation on the management of Indonesian nurses’ migration. This study aimed to monitor the implementation of the Code on state policy changing in facilitating nurse migration.

## Materials and methods

Qualitative and quantitative data were collected in order to understand the impact of the Code on Indonesian nurse migration. A triangulation approach was achieved through semi-structured interviews with key stakeholders, and records review of nurses’ migration in the last two years.

## Results

The Global Code of Practice contributed to shape the migration policy at the national level. This regulation provided a shift change of migration policy, which can be conducted by a country that had an agreement with Indonesia or a country that had a law on migrant protection. Acknowledging the importance of the Code, the MoH translated the Code into Indonesian, and disseminated the material to multiple stakeholders. By the spirit of this Code, Indonesia received financial and technical cooperation and agreement with Japan on the improvement of nursing capacity. The challenge faced by the MoH was a need for strong regulation which could accommodate the relevant players to coordinate on the national level, notably for the MoH, National Board for The Placement and Protection of Indonesia Manpower, Ministry of Foreign Affairs and private recruiters. Quantitative data showed that there was a significant flow of nurse migration, especially on nurses’ movement before and after the code was adopted. Nurse migration was increased four-fold between 2010 (567 nurses) to 2012 (2512 nurses) compared to three years before the Code was adopted. Indonesia’s government should carefully assess the flow of migration as the country has suffered a shortage of nurses. Lack of HRH information system and no integrated national HRH observatory hinder the policy maker to promote a strategic approach in nurse migration.

## Conclusions

The Code has been utilized by the Ministry of Health to manage migration. This guideline at the least provides direction that may be used where appropriate in the formulation and implementation of nurse migration. A stronger regulation which not only ties the MoH, but also other stakeholders in health migrant placement needs to be established. Further, strengthening HRH information system and research on the impact of migration on Indonesia’s health system must be conducted soon.

